# Obstetric and Neonatal Outcomes Associated With Implementation of a COVID-19 Predelivery Screening Policy in a Taiwan Hospital

**DOI:** 10.1001/jamanetworkopen.2023.3367

**Published:** 2023-03-16

**Authors:** Hui-Chin Lai, Po-Kai Juan, Szu-Ling Chang, Jen-Fu Tsai, Yun-Han Su, Hsiu-Wei Su

**Affiliations:** 1 Department of Anesthesiology, Taichung Veterans General Hospital, Taichung, Taiwan; 2 Department of Medicine, National Yang-Ming-Chiao-Tung University School of Medicine, Taipei, Taiwan; 3 National Chung-Hsin University, Taichung, Taiwan; 4 Department of Obstetrics and Gynecology, Wuri Lin Shin Hospital, Taichung, Taiwan; 5 Institute of Emergency and Critical Care Medicine, National Yang-Ming-Chiao-Tung University School of Medicine, Taipei, Taiwan; 6 Department of Obstetrics, Gynecology, and Women’s Health, Taichung Veterans General Hospital, Taichung, Taiwan

## Abstract

This cohort study examines obstetric and neonatal outcomes associated with predelivery screening policy implementation aimed to prevent COVID-19 in a Taiwan hospital.

## Introduction

In response to the COVID-19 pandemic, obstetric care practitioners worldwide have implemented preventive strategies such as universal or risk-based screening, restrictions on support persons, a designated environment for labor and childbirth, and adjustment of obstetric analgesia.^1^ Previous studies show that the COVID-19 pandemic, combined with disease prevention measures, has resulted in substantial changes in obstetric practice and outcomes.^2,3,4,5,6^ To our knowledge, no studies have demonstrated outcomes resulting from these antiepidemic policies alone.

In Taiwan, the Taichung Veterans General Hospital implemented a predelivery screening policy in May 2021. This policy required all women hospitalized for childbirth to provide a negative SARS-CoV-2 reverse transcriptase–polymerase chain reaction (RT-PCR) test result. Otherwise, patients were triaged in a negative-pressure room and were denied epidural anesthesia until a negative RT-PCR result was confirmed, which took at least 4 hours at that time. Consequently, many patients chose elective labor induction, as they could obtain a RT-PCR test result before their scheduled admission.

The time between predelivery screening policy implementation and first admission of a patient with confirmed SARS-CoV-2 infection enabled us to investigate outcomes of the policy alone. We aimed to assess the association of obstetric and neonatal outcomes with COVID-19 predelivery screening policy, and we hypothesized that outcomes before and after implementation were similar.

## Methods

The Taichung Veterans General Hospital Institutional Review Board approved this retrospective cohort study. Informed consent was not required because deidentified patient data were used. We followed the STROBE reporting guideline.

We reviewed the medical records of pregnant women (gestational age >36 weeks) hospitalized for childbirth between May 17 and July 10, 2021. We compared their maternal characteristics, obstetric outcomes, and neonatal outcomes with those of our historical control group, which comprised women hospitalized for childbirth between May 15 and July 10, 2020. We excluded pregnant women with any maternal or fetal conditions that precluded vaginal delivery.

Statistical analyses were performed with SPSS, version 24 (IBM SPSS). All analyses were 2 tailed, and *P* < .05 was considered statistically significant. Statistical analysis was performed on November 1, 2021.

## Results

This cohort study included 252 women (128 and 124 in the study and control groups; Table 1). Their mean (SD) maternal age was 33.2 (4.6) years; 77 study participants (60.2%) and 67 control participants (54.0%) were nulliparous. The prevalence of gestational diabetes was greater for study participants compared with control participants (12 [9.4%] vs 2 [1.6%]; odds ratio [OR], 6.26 [95% CI, 1.37-28.57]; *P* = .007).

**Table 1.** Participant Characteristics

Characteristic^a^	Control group (n = 124)	Study group (n = 128)	Mean difference	OR (95% CI)	*P* value
Age, mean (SD), y	33.2 (4.6)	33.2 (4.6)	−0.02	NA	.93
BMI, mean (SD)	26.5 (3.2)	26.8 (4.3)	0.48	NA	.74
Nulliparity	67 (54.0)	77 (60.2)	NA	1.28 (0.78-2.12)	.33
Gestational diabetes	2 (1.6)	12 (9.4)	NA	6.26 (1.37-28.57)	.007
Preeclampsia	2 (1.6)	2 (1.6)	NA	0.96 (0.13-6.93)	.97

Abbreviations: BMI, body mass index (calculated as weight in kilograms divided by height in meters squared); NA, not applicable; OR, odds ratio.

^a^ Unless noted otherwise, data are expressed as No. (%) of patients.

A total of 65 study participants (50.8%) received elective labor induction compared with 17 control participants (13.7%) (OR, 6.49 [95% CI, 3.50-12.05]; *P* < .001) (Table 2). The mean (SD) gestational age at childbirth was earlier for the study group compared with the control group (38.4 [1.4] vs 39.1 [1.0] weeks; *P* < .001). No differences in rates of cesarean section or assisted delivery were observed.

**Table 2.** Obstetric and Neonatal Outcomes

Outcome^a^	Control group (n = 124)	Study group (n = 128)	Mean difference	OR (95% CI)	*P* value	Adjusted OR (95% CI)^b^
Obstetric						
Gestational age, mean (SD), wk	39.1 (1.0)	38.4 (1.4)	0.62	NA	<.001	0.54 (0.84-0.25)
Labor stage, mean (SD), h						
First	6.3 (6.8)	6.7 (7.3)	0.38	NA	.61	NA
Second	1.4 (1.7)	1.2 (1.4)	0.25	NA	.56	NA
Elective induction	17 (13.7)	65 (50.8)	NA	6.49 (3.50-12.05)	<.001	NA
Epidural analgesia	97 (78.2)	106 (82.8)	NA	1.34 (0.72-2.51)	.36	NA
Delivery mode						
Cesarean section	21 (17)	22 (17)	NA	1.02 (0.53-1.96)	.96	1.03 (0.53-1.99)
Vacuum extraction	8(7)	10 (8)	NA			
Neonatal						
Birth weight, mean (SD), g	3054 (379)	3013 (385)	41.2	NA	.52	NA
Apgar score (5 min)						
Mean (SD)	8.9 (0.7)	8.9 (1.0)	0.04	NA	.83	NA
<7	3 (2.4)	3 (2.3)	NA	0.97 (0.19-4.89)	.97	NA
NICU admission	8 (6.5)	20 (15.7)	NA	2.71 (1.15-6.41)	.02	2.69 (1.13-6.42)

Abbreviations: NA, not applicable; NICU, neonatal intensive care unit; OR, odds ratio.

^a^ Unless noted otherwise, data are expressed as No. (%) of patients.

^b^ Adjusted for gestational diabetes.

More newborns in the study group were admitted to the neonatal intensive care unit (NICU) compared with newborns in the control group (20 [15.7%] vs 8 [6.6%]; OR, 2.71 [95% CI, 1.15-6.41]; *P* = .02). Their birth weights and Apgar scores were similar.

Multivariate analyses showed that elective induction, rather than gestational diabetes, was the main contributing factor for earlier gestational age at childbirth (mean difference, 0.89 [95% CI, 0.58-1.20]; *P* < .001) and increased NICU admission (adjusted OR, 2.69 [95% CI, 1.13-6.42]; *P* = .03).

## Discussion

The findings of this cohort study suggest that antiepidemic policies on hospitalization, elective labor induction, and epidural injection resulted in noticeable changes in obstetric practice. We observed that more women underwent elective labor induction; their newborns tended to be born earlier and were more likely to require intensive care. Limitations of this study include its retrospective design and generalizability of the findings. Our results are unlikely to be reproducible in regions with differences in antiepidemic policies, women’s attitudes and behaviors toward childbirth, and prepandemic obstetric practices. Nevertheless, we observed worsened maternal and neonatal outcomes associated with antiepidemic policies in some participants, and we encourage others to reexamine their policies.

## Notes

Supplement.
Data Sharing Statement
