# Supplementary material for: Obstetric and Neonatal Outcomes Associated With Implementation of a COVID-19 Predelivery Screening Policy in a Taiwan Hospital
Source: JAMA Netw Open. 2023 Mar 16;6(3):e233367. doi: 10.1001/jamanetworkopen.2023.3367 (PMC10020876; doi:10.1001/jamanetworkopen.2023.3367)
Supplement: Supplement. — Data Sharing Statement [file jamanetwopen-e233367-s001.pdf]

Data Sharing Statement

Lai. Obstetric and Neonatal Outcomes Associated With Implementation of a COVID-19 Predelivery Screening Policy in a Taiwan Hospital. *JAMA Netw Open*. Published March 16, 2023. doi:10.1001/jamanetworkopen.2023.3367

Data
Data available: No
